# Extracellular Vesicles Derived from *L-MYC* Neural Stem Cells Mediate Neuroprotection in 3D Models of Chemotherapy- and Radiation-Induced Neurotoxicity

**DOI:** 10.64898/2026.08.26.747380

**Published:** 2026-10-22

**Authors:** Lance G. A. Nunes, Isabella Vasquez, Brecken Enright, Liv Chen, Sunita K. Patel, Russell C. Rockne, Stephanie Yoon, Margarita Gutova

## Abstract

**Background/Objectives:**

Cancer survivors frequently experience long-term neurocognitive impairments following chemotherapy and cranial irradiation, yet experimental models that enable mechanistic investigation of therapy-induced neurotoxicity at the transcriptional level remain limited. This study aimed to develop a human three-dimensional (3D) neural tissue model derived from L-MYC-immortalized neural stem cells (LMNSCs) and use transcriptomic profiling to identify molecular pathways underlying chemotherapy- and radiation-induced neural injury and extracellular vesicle (EV)-mediated recovery.

**Methods:**

LMNSCs were differentiated in a 3D, methylcellulose-based culture to generate neural tissue containing neurons, astrocytes, and oligodendrocytes in the absence of microglia. Cultures were exposed to methotrexate (MTX) or ionizing radiation to induce neural injury and subsequently treated with LMNSC-derived EVs. Neural injury and repair mechanisms were evaluated by immunocytochemistry and bulk transcriptomics.

**Results:**

MTX and irradiation induced dose-dependent injury, exhibited by loss of neuronal complexity and reduced glial populations. LMNSC-EV treatment promoted recovery of neuronal and glial populations following MTX- and irradiation-induced injury. Transcriptomic analysis of irradiated cultures revealed activation of inflammation, DNA damage, and stress-response pathways, which were attenuated after treatment with LMNSC-EVs.

**Conclusions:**

LMNSC-based 3D neural tissue provides a platform for modeling cancer therapy-induced neurotoxicity. Furthermore, LMNSC-EVs represent a promising cell-free regenerative therapeutic that modulates injury-associated inflammatory, stress, and metabolic transcriptional programs after radiation-induced neural injury.

**Simple Summary:**

Many cancer survivors experience persistent problems with memory, attention, and learning after chemotherapy or radiotherapy, yet the biological mechanisms underlying these cognitive side effects remain poorly understood. Progress has been limited by the lack of human laboratory models that accurately reproduce treatment-induced brain injury. To address this need, we developed a three-dimensional human neural tissue model containing immature neurons, astrocytes, and oligodendrocytes derived from L-MYC-immortalized human neural stem cells, lacking microglia. This platform was used to model cancer therapy-induced neural injury and evaluate the regenerative potential of neural stem cell-derived extracellular vesicles (EVs)—small bioactive particles released by stem cells. We found that cancer therapies caused significant injury to neuronal, oligodendroglial, and astrocytic cell populations, whereas EV treatment promoted recovery of these cells and activated biological pathways associated with tissue repair. These findings establish a reproducible human neural tissue platform for studying cancer therapy-related neurotoxicity and support neural stem cell-derived EVs as a promising regenerative therapeutic strategy to prevent or reduce treatment-induced cognitive impairment.

